# Cerebrospinal fluid markers before and after shunting in patients with secondary and idiopathic normal pressure hydrocephalus

**DOI:** 10.1186/1743-8454-5-9

**Published:** 2008-04-25

**Authors:** Mats Tullberg, Kaj Blennow, Jan-Eric Månsson, Pam Fredman, Magnus Tisell, Carsten Wikkelsö

**Affiliations:** 1 Institute of Neuroscience and Physiology, The Sahlgrenska Academy at Göteborg University, Sahlgrenska University Hospital, SE 413 45 Göteborg, Sweden

## Abstract

**Background:**

The aim of this study was to explore biochemical changes in the cerebrospinal fluid (CSF) induced by shunt surgery and the relationship between these changes and clinical improvement.

**Methods:**

We measured clinical symptoms and analysed lumbar CSF for protein content, neurodegeneration and neurotransmission markers in patients with secondary (SNPH, n = 17) and idiopathic NPH (INPH, n = 18) before and 3 months after shunt surgery. Patients were divided into groups according to whether or not there was improvement in clinical symptoms after surgery.

**Results:**

Preoperatively, the only pathological findings were elevated neurofilament protein (NFL), significantly more so in the SNPH patients than in the INPH patients, and elevated albumin content. Higher levels of NFL correlated with worse gait, balance, wakefulness and neuropsychological performance. Preoperatively, no differences were seen in any of the CSF biomarkers between patients that improved after surgery and those that did not improve. Postoperatively, a greater improvement in gait and balance performance correlated with a more pronounced reduction in NFL. Levels of albumin, albumin ratio, neuropeptide Y, vasoactive intestinal peptide and ganglioside GD3 increased significantly after shunting in both groups. In addition, Gamma amino butyric acid increased significantly in SNPH and tau in INPH.

**Conclusion:**

We conclude that a number of biochemical changes occur after shunt surgery, but there are no marked differences between the SNPH and INPH patients. The results indicate that NFL may be a marker that can predict a surgically reversible state in NPH.

## Background

Normal pressure hydrocephalus (NPH) is a common and treatable cause of cognitive impairment and gait disturbance in the elderly[1]. NPH is generally thought of as a disorder resulting from disturbed cerebrospinal fluid (CSF) dynamics. Cerebral blood flow (CBF) is reduced in white and gray matter regions [2-5]. Microdialysis studies indicate compromised metabolism in the periventricular region [6]. Magnetic resonance (MR) images showing periventricular white matter lesions (WML) are a hallmark of NPH, the extent of which correlate with symptomathology [7-9]. After surgery, patients show increased CBF and reductions in periventricular WML in parallel with clinical improvement that are compatible with a restitution of brain function [2-5,8,9].

Previous studies on CSF composition in NPH patients have shown a variety of changes that indicate astrogliosis [10,11], axonal degeneration [11], inflammation [12] but no major demyelination [13]. Peptidergic neurotransmission is disturbed [14-16], whereas monoamine metabolites remain normal [13,17]. In a recent paper with the same group of patients [18], we studied the relationship between biomarkers in ventricular CSF and periventricular MRI pathology and explored concentration gradients of these markers between the ventricular and lumbar CSF. A correlation was found between the extent of periventricular WML and neurofilament protein (NFL) concentration in ventricular CSF, which corroborates a previous study on lumbar CSF [9].

There is considerable interest in investigating the diagnostic and predictive value of CSF markers in NPH. In a previous study, we reported that high preoperative CSF levels of the axonal marker, NFL correlated with the severity of symptoms and also with a favourable outcome after surgery indicating that the symptoms in NPH are associated with an ongoing but reversible axonal dysfunction [11]. This view is further supported by the correlations found between high CSF NFL, extended periventricular white matter lesions and pronounced symptoms in NPH patients [8,9,18]. To what degree the altered CNS metabolism and CSF dynamics induced by shunt surgery can influence the concentrations of axonal markers has not yet been studied to our knowledge. It is reasonable to predict that the increased levels of neuronal CSF markers reported in NPH decrease towards normal in parallel with clinical improvement, following shunt surgery. Further, it could be hypothesized that concentrations of neurotransmitter metabolites are associated with severity of symptoms.

The aim of the present study was to investigate in both SNPH and INPH patients 1) the preoperative levels of lumbar CSF markers reflecting neurodegeneration and neurotransmission, and clinical improvement after surgery; 2) the changes in lumbar CSF marker concentrations induced by surgery; and 3) correlations between lumbar CSF changes and clinical improvement after surgery. The patients included in this study are the same as in a previous paper [18] which examined ventricular CSF concentrations and white matter pathology before and after shunt surgery and the relationship between lumbar and ventricular CSF markers.

## Methods

### Patients

Thirty-five consecutive patients diagnosed with NPH at the Hydrocephalus Research Unit, Sahlgrenska University Hospital, Goteborg, Sweden, and who had a repeat CSF examination were included (table 1). These are the same patients as reported in a previous paper [18]. All patients received a ventriculo-peritoneal shunt: a Sophy™ Programmable Pressure Valve (Sophysa, Orsay Cedex, France) or a Delta Valve (Medtronic PS Medical, Santa Barbara, USA). The Göteborg University Ethics Committee approved the study and verbal informed consent was obtained from all patients.

**Table 1:** Demographic features of patients.

	Secondary NPH (n = 17) Mean (SD), frequency	Idiopathic NPH (n = 18) Mean (SD), frequency
Age (years)	65 (10); range 49–79	68 (15.7); range 19–86
Males/Females	12/5	11/7
Duration of symptoms (months)	16 (19); range 2–60	40 (37); range 11–164
**Etiology**		
Subarachnoid hemorrhage	9 (53%)	
Cerebrovascular disorder	4 (23%)	
Trauma	2 (12%)	
Other*	2 (12%)	
Cerebrovascular risk factors		
Hypertension	4 (23%)	5 (28%)
Cardiovascular disorder	3 (18%)	5 (28%)
Diabetes	1 (6%)	2 (11%)

Cerebrovascular disorder etiology was considered when there was a temporal relationship between an ischemic or hemorrhagic incident other than subarachnoid hemorrhage and development of NPH symptoms. * Meningioma (n = 1) and Recklinghausen's disease (n = 1). These are the same patients as reported in a previous paper [18].

### Study protocol

Diagnostic criteria for NPH have been described previously [8] and were a) gait disturbance; b) mental deterioration, or urinary incontinence, or both; c) enlarged ventricles on computerised tomography (CT) or MRI with Evan's index > 0.30; d) a lumbar CSF pressure < 20 cm H_2_O; e) ventricular filling and block of convexity flow on radionuclide cisternography (RC). The CSF tap test [19] and regional cerebral blood flow (rCBF) measurement was performed on some patients with signs of other disorders, such as vascular lesions. Only patients with improvement at the CSF tap test and a characteristic pattern on rCBF (n = 11) [2] were included. The probable cause of the NPH state was assessed and patients were diagnosed with idiopathic etiology (INPH) if no known cause could be identified. Three months postoperatively, a re-examination was performed using the same protocol except RC. If a patient had not improved clinically and the ventricular system had not decreased in size at the re-examination, the shunt was tested by radionuclide shuntography and was surgically corrected if malfunctioning. Using the clinical measures described below, patients were divided into two groups for data analysis: those improved by shunt surgery or those unimproved by surgery.

### Clinical measures

Patients were evaluated before and three months after surgery according to a previously published study protocol [13,20,21] comprising a semi quantitative evaluation of twelve symptoms and signs measuring gait, balance, mental and bladder performance. Mini-Mental State Examination (MMSE) was used to assess global psychometric performance. Simple reaction time was calculated as the median of 10 tests using simultaneous sound and light stimuli. The other psychometric tests used were the identical forms test (perceptual speed and accuracy) and Bingley's test (learning and memory) [11]. Bingley's visual recognition test was performed by presenting a picture of 12 drawings of familiar objects over a 30 second period. Recognition was tested immediately. Each test was done twice and the mean calculated. The impairment of wakefulness was assessed as presence of somnolence-sopor-coma disorder (SSCD) based on the original coarse division of SSCD into a mild, a moderate and a severe form by Lindquist and Malmgren [22]. Clinical indices reflecting global, psychometric, balance, gait and continence performance were calculated as previously described [20] (Table 2; see Statistics).

**Table 2:** Clinical characteristics of SNPH and INPH patients pre- and postoperatively.

**Index**	**Function/Test**	**Preop SNPH (n = 17)**	**Preop INPH (n = 18)**	**Postop SNPH (n = 17)**	**Postop INPH (n = 18)**
**Global**	MMSE (0–30)	17.2 (9.6)	23.3 (7.4) *	23.3 (4.6) **	25.9 (7.9) **
**Psycho-metric**	Identical forms test (0–60)	14.4 (17.8)	24.4 (12.3)	20.9 (17.3) ns	32.3 (12.1) ns
	Bingley's test (0–12)	1.9 (2.1)	4.0 (2.1) *	3.9 (1.7) **	4.4 (1.8) ns
	Reaction time test (s)	1.7 (3.0)	1.4 (1.8)	0.5 (0.7) ns	0.9 (1.3) ns
**Balance**	Romberg's test (0–60)	24.9 (27.2)	42.8 (26.1)	41.1 (25.5) **	50.4 (22.0) **
**Gait**	Gait performance (1–6)	3.4 (1.8)	2.4 (1.2) *	2.2 (1.5) **	1.8 (1.1) ns
	Time needed to walk 10 m (s)	38.5 (43.1)	23.4 (35.3)	11.4 (2.2) *	12.6 (4.2) *
	Steps needed to walk 10 m (#)	42.8 (38.1)	30.1 (22.4)	19.9 (3.7) **	19.5 (4.6) **
**Cont-inence**	Urgency incontinence (yes/no)	4/11	8/10 *	11/5 *	5/13 ns
**Wake-fulness**	IW (1/2/3/4)	4/7/3/3	9/7/2 *	14/3/0/0 **	14/4/0/0 *
**MoD**				0.75 (0.72)	0.33 (0.37)

Values are given as Mean (SD) or frequency. Gait performance is registered as 1–6: 1 = normal; 2 = insecure; 3 = insecure cane; 4 = bimanual support; 5 = aided; 6 = wheelchair. 1–4: 1 = none; 2 = slight; 3 = moderate; 4 = severe. IW = Impairment of wakefulness (1 = none; 2 = slight; 3 = moderate; 4 = severe). MoD = mean of differences which is a measure of overall improvement (see statistics). Significant differences between SNPH and INPH patients preoperatively are indicated in the Preop INPH column. Differences between the pre- and postoperative assessment were analysed for SNPH and INPH patients as indicated in the Postop columns. ns = non significant; * = *p *≤ 0.05; ** = *p *≤ 0.01; *** = *p *≤ 0.001.

### CSF analysis

Lumbar puncture (LP) was performed in the L3/L4 or L4/L5 interspace with the patient in a recumbent position on the morning before, and three months after shunt surgery. The CSF pressure was measured and the first 12 ml of CSF were collected in ice-chilled tubes, aliquoted, centrifuged and frozen at -80° until analyzed.

The CSF concentrations of the major monoamine metabolites, homovanillic acid (HVA), 5-hydroxy-indoleacetic acid (5-HIAA) and 4-hydroxy-3-methoxyphenylglycol (HMPG) [23], sulphatide [24], neuropeptide Y (NPY) [25], vasoactive intestinal peptide (VIP) [14], tau protein [26], ganglioside GD3 (GD3) [27], gamma amino butyric acid (GABA) [13] and neurofilament protein (NFL) [28] were determined as previously described. Albumin in serum and CSF was determined by nephelometry. The CSF/serum albumin ratio [29] was used as a measure of the blood-brain barrier (BBB) integrity [29].

### Statistics

Nonparametric Mann-Whitney U test was used for between group comparisons. Spearman correlation coefficients were calculated in correlation analyses. Changes between pre- and postoperative values were analyzed by Wilcoxon signed rank sum test. The clinical indices were constructed by calculating the mean of the variables included in each index (Table 2). Scores were standardized to enable comparisons across indices [20]: preoperative variables were z-standardized (mean of 0 and a standard deviation of 1.0) and postoperative variables were standardized using the mean and SD of the preoperative variables. The difference between the pre- and postoperative values was calculated for each index, and the mean of the differences (MoD) was calculated as the overall result after shunt surgery. Patients showing MoD > 0.05 were considered improved, which corresponded to the clinical impression in each case. A *p*-value < 0.05 was considered significant.

## Results

### Clinical measures

Clinical assessments of patient groups are given in Table 2. Preoperatively, the INPH patients were significantly better than the SNPH patients for MMSE, Bingley's test, gait performance and wakefulness but were significantly worse for continence.

After shunt surgery, 28 patients (80%) were improved (MoD 0.12 to 2.4), 5 (14%) were unchanged (MoD -0.04 to 0.04) and 2 (6%) deteriorated (MoD -0.19 and -0.45, data not shown). Fifteen of the SNPH patients were improved, whereas 2 deteriorated (MoD -0.19 to -0.45). Thirteen (72%) of the INPH patients improved, whereas 5 (28%) were unchanged (MoD -0.03 to 0.03, data not shown). The overall improvement (MoD) for all patients was 0.53 ± 0.60 (mean ± SD) without any significant gender differences (data not shown). Secondary NPH patients (MoD = 0.75 ± 0.72) showed more overall improvement than INPH patients, but the difference was not significant (MoD = 0.33 ± 0.37), Table 2. Specifically, eight tests were significantly improved in the SNPH patient compared to five in the NPH patients.

### CSF biomarkers pre- and postoperatively

Preoperatively in both groups, the only pathological findings were elevated neurofilament protein (NFL) and elevated albumin (Table 3). SNPH patients also had an elevated albumin ratio. Apart from this, the only difference seen between SNPH and INPH patients was in NFL, which was higher in secondary cases (4884 ± 5855 vs 1352 ± 2989 ng/L, *p *= 0.01, Table 3). Preoperatively, there were no differences in any CSF biomarker between patients that improved and those that did not improve after surgery. Patients with cerebrovascular etiology (n = 4) versus those without, had higher sulphatide (287.5 ± 85.4 vs 197.2 ± 97.2 nmol/l; n.s.) and albumin ratios (12.3 ± 2.9 vs 9.9 ± 8.6; *p *= 0.09, data not shown).

**Table 3:** Marker concentrations in lumbar CSF pre- and postoperatively.

		**CSF preoperatively**				**CSF postoperatively**			
	**Lab reference value**	Preop SNPH (n = 17)	Preop INPH (n = 18)	Preop Improved (n = 28)	Preop Unimproved (n = 7)	Postop SNPH (n = 17)	Postop INPH (n = 18)	Postop Improved (n = 28)	Postop Un-improved (n = 7)

**Protein content:**									
Albumin (mg/L)	< 400	710 (511) pathol	544 (171) ns	650 (413)	529 (211) ns	1522 (3044) ↑ ***	1217 (1052) ↑ ***	1439 (2569) ↑ ***	1141 (935) ns
Albumin ratio	< 10.2	12.2 (11.0) pathol	8.4 (3.7) ns	10.8 (8.9)	8.0 (4.6) ns	24 (46) ↑ ***	21 (22) ↑ ***	24 (40) ↑ ***	19 (17) ns
**Transmitter function:**									
HVA (nmol/L)	253 (109) [23]	209 (146) normal	225 (155) ns	240 (154)	140 (102) ns	229 (133) ns	218 (79) ns	230 (111) ns	202 (112) ns
5-HIAA (nmol/L)	125 (54) [23]	111 (56) normal	118 (95) ns	126 (80)	74 (53) ns	119 (50) ns	123 (33) ns	127 (42) ns	96 (38) ns
HMPG (nmol/L)	47 (10) [23]	37 (15) normal	36 (16) ns	39 (16)	28 (13) ns	38 (9) ns	44 (16) ns	43 (13) ns	31 (7) ns
NPY (pmol/L)	120–170 [25]	89 (26) low	106 (36) ns	95 (30)	108 (39) ns	110 (31) ↑ ***	155 (46) ↑ ***	124 (38) ↑ ***	161 (60) ns
VIP (pmol/L)	< 20 [14]	6.1 (1.7) normal	8.4 (4.9) ns	6.4 (1.4)	10.5 (7.9) ns	13 (5) ↑ ***	26 (22) ↑ **	16 (5) ↑ ***	36 (38) ns
GABA (nmol/L)	50–130 [36]	53 (30) normal	67 (44) ns	53 (25)	80 (58) ns	84 (64) ↑ **	103 (115) ns	91 (100) ↑ *	103 (25) ns
**Demyelination:**									
Sulphatide (nmol/L)	< 300 [24]	210 (111) normal	208 (91) ns	210 (100)	204 (105) ns	239 (116) ns	360 (318) ns	279 (245) ns	363 (195) ns
**Gliosis:**									
GD3 (nmol/L)	50 (12) [27]	39 (25) normal	38 (22) ns	35 (22)	50 (25) ns	56 (28) ↑ *	70 (33) ↑ ***	59 (32) ↑ **	79 (20) ns
**Neuronal degeneration:**									
NFL (ng/L)	< 300 [28]	4884 (5855) pathol	1352 (2989) **	3637 (5338)	938 (1102) ns	3178 (4315) ns	1828 (3313) ns	2647 (3550)	2161 (1815) ns
TAU (ng/L)	< 400 [26]	311 (354) normal	229 (160) ns	267 (296)	275 (165) ns	341 (174) ns	651 (256) ↑ ***	449 (248)	633 (305) ns

Values are given as mean (SD). Laboratory reference values are given in Column 1 where superscript numbers in square parentheses are literature references to laboratory reference values. INPH patients had significantly lower preoperative NFL than SNPH patients, as indicated in the preoperative INPH column, otherwise no differences were seen between groups preoperatively. There were no differences in preoperative CSF concentrations between improved and unimproved patients as indicated in the preoperative unimproved column. NFL was numerically, but not significantly, higher in the preoperative improved than in the unimproved group. There were no differences in postoperative CSF concentrations between improved and unimproved patients as indicated in the postoperative unimproved column. Postoperative values compared to preoperative levels are indicated in the postoperative columns. A number of markers showed significant increases postoperatively in the SNPH, INPH and improved groups as indicated by arrows. ns = non significant; * = p ≤ 0.05; ** = p ≤ 0.01; *** = p ≤ 0.001. The data for improved and unimproved patients has been published previously [18].

After surgery, significant increases were seen in both SNPH and INPH groups for albumin, albumin ratio, NPY, VIP, GD3. On the other hand, HVA, HMPG and 5-HIAA and sulphatide remained unchanged (Table 3). In addition, GABA was significantly increased in SNPH patients and tau in the INPH patients. These postoperative changes were significant also in the improved subgroup of patients but not in the unimproved subgroup. However, this could be due to fewer patients in the unimproved group (% change similar in both groups). NFL was numerically but non-significantly reduced in all patients, except for the unimproved group where there was a small increase.

### Association between clinical measures and CSF biomarkers

#### Preoperative measures

In the whole sample, higher NFL correlated significantly with poorer performance on a number of clinical tests (MMSE, Identical forms test, Bingley's test, Romberg's test, gait general, gait 10 m (number of steps), gait 10 m (speed), more pronounced impairment of wakefulness and poorer overall psychometric performance; r = 0.35–0.64, *p *= 0.0001–0.04). Higher preoperative NFL correlated with greater improvement in wakefulness (r = 0.46, *p *= 0.006) and non-significantly with better overall improvement (r = 0.31, *p *= 0.08). Higher NFL was registered in the 9 patients with the greatest postoperative improvement (upper 25^th ^percentile) than in the 9 patients with the worst postoperative outcome (lower 25^th ^percentile) (6415 ± 6253, mean ± SD, vs 772 ± 1010; *p *= 0.006, data not shown); otherwise no significant differences were seen between these two groups.

Higher tau correlated with a more pronounced impairment of wakefulness (r = 0.49, *p *= 0.001)) and higher albumin ratio correlated with worse psychometric performance (r = 0.49, *p *= 0.03); otherwise no significant correlations were seen between other biomarker concentrations and clinical tests.

#### Postoperative measures

**Figure 1:**
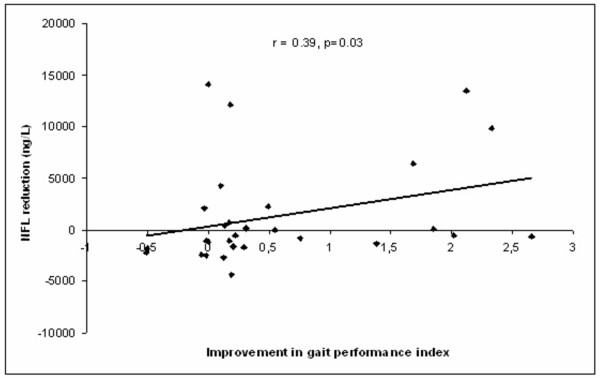
Scatterplot showing the relationship between postoperative reduction in NFL and improvement in gait performance index.

A more pronounced postoperative decrease in NFL correlated with greater improvement in a number of tests in both patient groups combined: gait 10 m (number of steps, r = 0.49, *p *= 0.02); Romberg's test (r = 0.41, *p *= 0.03); gait performance index (r = 0.39, *p *= 0.03, Figure 1). In INPH patients, the decrease in NFL did not correlate significantly with improvement for Rombergs test (r = 0.56) and for gait 10 m (number of steps, r = 0.55, *p *= 0.06).

A lower postoperative increase in tau correlated with greater improvement in the identical forms test (r = -0.88, *p *= 0.01) and MMSE (r = -0.45, *p *= 0.01). A lower postoperative increase in NPY correlated with greater improvement in MMSE (r = -0.52,*p *= 0.01), wakefulness (r = -0.45, *p *= 0.02), psychometric performance (r = -0.40, *p *= 0.04) and overall improvement (r = -0.45, *p *= 0.02). There was no correlation between postoperative changes in VIP, GABA, HVA, HMPG, 5-HIAA, sulphatide or GD3 and overall improvement after surgery, in either the total group or in the improved patients (r < 0.36, *p *> 0.11).

## Discussion

The main findings of this study were the correlation between 1) a higher preoperative NFL and more severe clinical symptoms and 2) a greater postoperative reduction in NFL and more pronounced clinical improvement. These findings corroborate previous reports [11,18], and give novel support to the view that the symptomatology in NPH is caused by a periventricular neuronal dysfunction that can be reversed by a shunt operation. Preoperatively, the most important pathological finding was elevated NFL in both idiopathic and secondary cases. Hypothetically, an altered periventricular microenvironment caused by CSF flow into the white matter could lead to an axonal/neuronal dysfunction, possibly through metabolic disturbance [6,30]. As a consequence, neurons may start to leak structural proteins, such as NFL, into the interstitial fluid and subsequently into the CSF. The higher NFL concentrations seen in the secondary cases are in accordance with the hypothetically more aggressive pathophysiological process in secondary NPH and the more pronounced improvement seen in these patients.

The findings of this study could be of clinical relevance for certain patient groups. Thus, a CSF pattern of elevated NFL but normal tau and sulphatide supports the possibility that in patients with dementia and gait disorder, there could be diagnostic discrimination between NPH and Binswanger's disease, when there is increased sulphatide [13], and Alzheimer's disease, when there is increased tau[26].

No baseline CSF marker could identify shunt responders with certainty, which indicates that the prognostic value of CSF marker analysis is limited in NPH. Consistent with a previous study [11], higher preoperative NFL concentrations were correlated with more favorable outcome after shunt surgery. However, it should be noted that there were patients in our sample with normal NFL who improved substantially.

Improved patients showed a postoperative reduction in NFL, although not significant, whereas unimproved patients showed a numerical increase in NFL. In the total sample, no significant reduction in NFL was seen. One reason for this could be a coexisting cerebrovascular disease in some patients causing NFL to remain elevated as signs of cerebrovascular disease are common in NPH [7,8,31,32]. Thus, the NFL increase could hypothetically originate from different pathophysiological processes: one related to CSF dynamic disturbance (NPH), another to ischemic white matter degeneration (cerebrovascular disease). Yet another reason could be that the follow-up period was too short, as NFL levels remain elevated in the CSF three months after an acute stroke episode (Lars Rosengren, personal communication).

We cannot rule out that changes in some biomarkers are related to the initial insult in SNPH patients. The time between the initial insult and shunting in SNPH patients was 2 months (n = 5) or = 4 months (n = 4). SNPH patients had higher NFL levels than did INPH patients, which favours this view. On the other hand, tau, another neuronal marker that shows increased concentrations after acute brain damage [33,34], did not differ between SNPH and INPH patients. It cannot be ruled out that hyperphosphorylated tau, which more specifically reflects neuronal damage than total tau measured here, could differ between the groups.

The SNPH and INPH patients were analyzed separately to allow for possible differences in pathophysiology and comorbidity between INPH and SNPH cases. The two groups showed a similar pattern of postoperative CSF marker changes. Correlations similar to those of the whole sample were observed in the INPH group between CSF marker concentrations and clinical measures.

A possible explanation for the postoperative biochemical changes shown in this study could be altered CSF dynamics related to shunt placement, i.e. increased CSF flow from the ventricles. However, a postoperative increase was seen for some CSF markers but not others (monoamine metabolites, sulphatide and NFL). This difference between different peptides could argue against this explanation since one would expect similar postoperative changes for all markers if changes were caused by altered CSF dynamics. Previously, it was shown that there is no association between ventricular size and CSF marker concentrations which argues against the assumption that variations in ventricular size explains differences in CSF marker concentrations [18]. Also, MRI studies show very rapid mixing of CSF within the subarachnoid space [35] which argues against gradients caused by the shunt device.

Due to the methodological problems mentioned, the biochemical alterations shown here should be interpreted with care. Our findings indicate that there is no major down regulation of neurotransmission in NPH as levels of monoamine metabolites and neuropeptides remain within normal limits both pre- and postoperatively. The postoperative increase seen for tau could possibly be due to a perioperative trauma to the cerebral cortex caused by the ventricular catheter. Accordingly, tau levels remain elevated up to five months after an acute stroke episode [33]. The postoperative increase in albumin ratio (blood brain barrier function), sulphatide (demyelination) and GD3 (astrogliosis) could indicate that shunt implantation may induce different pathological alterations in the brain. Future CSF studies of NPH patients with longer follow-up (12 months or more) are warranted to settle these issues.

## Conclusion

A number of biochemical changes occur in the lumbar CSF after shunt surgery, including both functional and structural biomarkers. The results of this study indicate that NFL could be a marker that reflects the reversible state in NPH. Higher NFL levels occurred in patients with greater impairment. Higher preoperative NFL levels were also correlated with more marked clinical and functional improvement after shunting. Additionally, a greater postoperative reduction in NFL correlated with more pronounced clinical improvement.

## Competing interests

The authors declare that they have no competing interests.

## Authors' contributions

All authors participated in the design of the study, data analysis and draft of the manuscript. MT examined the patients, performed LPs and was responsible for data handling and analysis and manuscript preparation. KB, J-EM and PF carried out the different CSF analyses. MTi, performed the shunt operations. CW examined patients, performed LPs and participated in data analysis. All authors read and approved the final manuscript.
